# Reasons for continued usage of ENDS: Differences by device and liquid characteristics among US adults

**DOI:** 10.18332/tpc/189924

**Published:** 2024-06-21

**Authors:** Qinghua Nian, Jeffrey J. Hardesty, Elizabeth Crespi, Joanna E. Cohen

**Affiliations:** 1 Institute for Global Tobacco Control, Department of Health, Behavior and Society, Johns Hopkins Bloomberg School of Public Health, Johns Hopkins University, Baltimore, United States

**Keywords:** addiction, smoking cessation, electronic nicotine delivery systems, reasons to use

## Abstract

**INTRODUCTION:**

Motivations for using electronic nicotine delivery systems (ENDS) include quitting or reducing cigarette smoking, flavor, and addiction. This study examines whether the primary reason for continued ENDS usage changes over time, and its association with device and liquid characteristics.

**METHODS:**

Data are from a longitudinal cohort study and include 526 US adults (≥21 years) using ENDS frequently (≥5 days/week) as self-reported, and uploaded photos of their most used ENDS devices and liquids and self-reported primary reason for continued ENDS usage in wave 2 (December 2020–April 2021) and wave 5 (February–April 2023). Device-liquid grouping was defined by device (disposable/disposable pod/refillable pod/tank, adjustable/no adjustable settings) and liquid (salt/freebase) characteristics. A device was classified as having adjustable settings if it allowed users to modify the power, coil, or airflow. Data were analyzed using multivariable logistic regressions and McNemar tests.

**RESULTS:**

From wave 2 to 5, the primary reason for continued ENDS usage significantly changed, with more participants reporting addiction (29.2% vs 34.6%, p<0.001); and significantly more participants used disposable devices (salt, no adjustable settings) (7.9% vs 25.2%, p<0.001). Compared to those using tanks (freebase, adjustable settings), participants using devices with nicotine salt liquids were more likely to report addiction (AOR>2; 95% CI: 1.12–8.19); and participants using disposable devices (salt, no adjustable settings) were less likely to report quitting/reducing smoking as the primary ENDS use reason after controlling for smoking status and sociodemographic characteristics (AOR<0.6; 95% CI: 0.14–0.995).

**CONCLUSIONS:**

Over a 2.5-year period, the proportion of participants continuing to use ENDS at least 5 days/week because of addiction grew, and participants’ motivations varied by device-liquid grouping. Restrictions on nicotine salts may disproportionately impact those using ENDS because of addiction; and regulations targeting tanks with freebase liquids may disproportionately impact those using ENDS for smoking cessation/reduction.

## INTRODUCTION

About 6.9% of US adults aged ≥18 years currently use electronic nicotine delivery systems (ENDS), and 3.2% use ENDS daily^1^. ENDS typically include coils powered by batteries, which heat and aerosolize liquids containing nicotine, propylene glycol and/or vegetable glycerin, and various flavors^2^; and are available with many devices (e.g. dimensions of refillability, device liquid container, setting adjustability) and liquid characteristics (e.g. nicotine concentration, freebase vs nicotine salt, propylene glycol/vegetable glycerin ratio)^3,4^. Over 80% of e-cigarettes sold in the US in 2022 have high nicotine concentrations (≥5%)^5^.

Adults are often motivated to use ENDS in order to quit or reduce smoking^6,7^. Berg et al.^7^ examined reasons for ENDS use among young adults aged 18–34 years in the US and found that quitting smoking was more frequently endorsed by people who formerly smoked than those who currently smoked or never smoked (85% vs 59% vs 44%, respectively, all p<0.001)^7^. People may also be motivated to use ENDS because of flavors. In a 2014 US study, approximately 60% of adults aged 18–34 years endorsed flavor as a reason for using ENDS, with no significant differences based on smoking status^8^. Several studies have compared motivations for ENDS use among both younger and older adults, finding that younger adults were more likely to use ENDS for reasons such as flavors or social reasons^9,10^ and less likely for cessation or reduction of smoking compared to older adults^10^.

Specific ENDS devices and liquid characteristics, such as nicotine content and delivery^7,11^ or sleek device designs and easy disposability^11-13^, may be associated with people’s motivations to use ENDS. In a 2018 study among US adults aged ≥18 years using devices shaped like USB flash drives, nicotine content was the primary reason (30.7%) to use ENDS, while attempting to quit other tobacco products (22.6%) was not the predominant reason^11^. However, there is limited knowledge about the association between the primary reason for using ENDS (such as smoking cessation, addiction) and the choice of ENDS device and liquid characteristics one uses.

Additionally, some studies explored affective and social motivations for ENDS use, such as deriving satisfaction and pleasure from ENDS use and seeking a larger social environment^14^. A US study observed significant decreases over a 12-month period in affect-related reasons (e.g. to relax, to increase positive feelings) for ENDS use among young adults aged 18–24 years and no significant change in social reasons (e.g. to feel part of a group, to celebrate)^15^. Beyond affective and social motivations, little is known about longitudinal changes in the primary reason for continued ENDS usage (e.g. switching from smoking cessation to flavors over the years); and whether the association with the choice of ENDS device and liquid characteristics evolves over time, given people may change their primary reason for ENDS use and the combinations of their ENDS device and liquid characteristics in a rapidly changing market.

This study focuses on adults using ENDS frequently. It fills this research gap by exploring the association between the primary reason for continuing to use ENDS (the main motivating factor or rationale that drives an individual to continue to use ENDS) and combinations of ENDS device type, device adjustability, and liquid formulation controlling for sociodemographic characteristics and smoking status; and observing changes in primary reason that are not limited to affective and social motivations among US adults frequently using ENDS from 2020 to 2023.

## METHODS

### Study sample and protocols

The Vaping and Patterns of E-cigarette Use Research (VAPER) study is a web-based longitudinal cohort study of adults (aged ≥21 years) in the US who used ENDS and ENNDS (Electronic Non-Nicotine Delivery Systems) at least five days/week^16^. Nearly all respondents (98%) reported using ENDS (containing nicotine). This study used data from waves 2 (December 2020–April 2021) and 5 (February–April 2023) of the VAPER study. Recruitment utilized a Craigslist-focused strategy, posting ads on gigs and job boards. Eligible participants provided verifiable identifying data, including name, date of birth, mailing address, and cell phone number; they also completed online surveys on REDCap, reporting their ENDS use patterns and behaviors. They also submitted photos of their most frequently used ENDS device and liquid. Survey responses were utilized when device and liquid characteristics were unavailable from photos. Rigorous data review and cleaning procedures were implemented after coding to ensure the data quality and authenticity of submitted photos. Detailed information about the protocols has been reported^16^. Follow-up survey invitations were sent to people’s mobile phones and email addresses, and the links to the survey were tied to their previously established record ID number if they validly responded and expressed interest in participating in subsequent waves. To maintain eligibility in follow-up waves, participants were required to report continued use of ENDS for at least five days per week.

The survey question regarding the primary reason for continuing to use ENDS was modified prior to wave 2. To examine the longitudinal change in primary reason for continued usage, we used data in wave 2 (December 2020–April 2021) as the 1st timepoint and wave 5 (February–April 2023) data as the 2nd timepoint in this study.

In wave 2, 1187 participants validly responded to the survey. Among these, 526 indicated an interest in staying in the study, reported still using ENDS ≥5 days per week, and provided valid responses in wave 5; this is the study sample. There was no significant difference between the study sample and other participants in wave 2 in terms of sociodemographic characteristics (including age, gender, race, income, region, and Hispanic origin) (p>0.05). The Virginia Commonwealth University Institutional Review Board (IRB) approved the study (HM20015004), with the Johns Hopkins Bloomberg School of Public Health IRB relying on the IRB of VCU as the IRB of record (IRB9277).

### Measurements

*Primary reason for continuing to use ENDS*

Participants were asked to select one reason as the primary reason for continuing to use ENDS from options: ‘To quit smoking’, ‘To cut down smoking’, ‘To use when I’m not allowed to smoke’, ‘Absence of smell’, ‘Because I enjoy the flavor’, ‘Because I enjoy the boost’, ‘Curiosity/Just want to try them’, ‘Because I am addicted’, and ‘Some other reason (including stress, anxiety, depression, or other mental health concerns, etc.)’. We classified those options into four categories: 1) quitting/reducing smoking (‘To quit smoking’, ‘To cut down smoking’); 2) addiction (‘Because I am addicted’); 3) flavor (‘Because I enjoy the flavor’); and 4) other reasons (‘To use when I’m not allowed to smoke’, ‘Absence of smell’, ‘Because I enjoy the boost’, ‘Curiosity/Just want to try them’, ‘Because of stress, anxiety, depression, or other mental health concerns’, and ‘Some other reason’), then created a dichotomous variable (1=Yes, 0=No) for each category. A sensitivity test showed that there was no significant difference between combining ‘enjoy the boost’ with ‘addiction’ and combining it with ‘other reasons’.

*Device-liquid grouping*

We classified ENDS by examining the combination of the device (reusability, refillability, uses a tank or pod, modifiability of the power or airflow or coil) and liquid (freebase and nicotine salt) characteristics that can affect user nicotine and toxicant exposure^3,17^. A device was classified as having adjustable settings if it allowed users to modify the power, coil, or airflow. We coded device and liquid information directly from submitted photos and searched the internet to identify the characteristics of the device (e.g. brand, model) and liquid (e.g. nicotine formulation and concentration). The self-report data were used when device or liquid characteristics were not available from submitted photos or internet searches. Over 90% of participants reported using one of five device-liquid groupings in waves 2 and 5: 1) disposable device (salt, no adjustable settings); 2) disposable pod (salt, no adjustable settings); 3) refillable pod (freebase, adjustable settings); 4) refillable pod (salt, adjustable settings); and 5) tank (freebase, adjustable settings).

*Other variables*

Participants self-reported their geographical location (Northeast, Midwest, South, West), gender (man, woman, non-man/woman, prefer not to answer), age (21–29, 30–44, ≥45 years), age of first use of ENDS (<21, ≥21 years), race (White, other single race, multi-racial, prefer not to answer), ethnicity (Hispanic/Latino, non-Hispanic/Latino, prefer not to answer), income ($) (0–39999, 40000–59999, 60000 –99999, ≥100000, prefer not to answer), and sexual identity (heterosexual or straight, non-heterosexual or non-straight, prefer not to answer). Smoking status was based on two items: 1) ‘How many cigarettes have you smoked in your entire life?’; and 2) ‘Have you smoked a cigarette in the past 30 days?’. Participants were classified as people who have never smoked if they smoked <100 cigarettes in their lifetime; as people who formerly smoked if they smoked ≥100 cigarettes in their lifetime and did not smoke in the past 30 days; and as people who currently smoke if they smoked ≥100 cigarettes in their lifetime and smoked in the past 30 days. ENDS dependence was measured by a 4-item E-cigarette Dependence Scale (EDS) score (range: 0–4, 4 being most dependent)^18^.

### Statistical analysis

All analyses were conducted using SAS (Version 9.4, SAS Institute, Cary, NC). Descriptive statistics were provided for sociodemographic variables, smoking status, and ENDS device-liquid grouping for the overall sample and by reasons for continued ENDS usage, in each wave. The Rao-Scott chi-squared test was used to assess differences in categorical variables, and the McNemar test was used to determine if the proportions of categories in the two waves differed significantly. Associations between primary reason for continued ENDS usage (quitting/reducing smoking, addiction, flavor, and other reasons) and device-liquid grouping, were determined through multivariable logistic regression models. Adjusted odds ratios (AORs) with 95% confidence intervals (CIs) were calculated and reported for independent variables. These models controlled for participants’ sociodemographic characteristics (age, race, and sexual orientation) and smoking status.

Based on data from the 2019 Tobacco Use Supplement to the Current Population Survey (TUS-CPS), post-stratification survey weighting was employed for gender, age, and race, with weights ranging from 0.33 to 1.80. This ensured the representativeness of US adults who frequently use ENDS. Weighted frequencies (n), scaled to our sample size, are reported for all analyses. P-values were adjusted to accommodate the large sample size resulting from applying survey weights. The pairwise deletion method handled missing data (<2% of participants). A 2-sided p<0.05 was considered statistical significance.

## RESULTS

Most participants (81.4%) were aged <45 years, non-Hispanic White (85.3%), and 37.2% had an annual income of less than $40000 (Table 1). About two-thirds of participants formerly smoked (W2: 64.6%; W5: 69.8%) and about 20% currently smoked (W2: 22.1%; W5: 18.2%) (Table 2). Compared to wave 2, a significantly higher percentage of participants used disposable devices (salt, no adjustable settings) (7.9% vs 25.2%, p<0.001).

**Table 1:** Sociodemographic characteristics, smoking status, and device-liquid grouping by primary reasons for continued ENDS usage, VAPER cohort study, wave 2 (December 2020–April 2021) and wave 5 (February–April 2023) (N=526)

		*Total n (col %)*	*W2: Reasons for continued ENDS usage*				*W5: Reasons for continued ENDS usage*			
			*Quitting/reducing smoking*	*Addiction*	*Flavor*	*Others*	*Quitting/reducing smoking*	*Addiction*	*Flavor*	*Others*
			*n (row %)*	*n (row %)*	*n (row %)*	*n (row %)*	*n (row %)*	*n (row %)*	*n (row %)*	*n (row %)*
**Total**		526 (100)	229 (43.5)	154 (29.2)	67 (12.8)	77 (14.6)	169 (32.1)	182 (34.6)	58 (11.1)	117 (22.2)
**Geographical location**	Northeast	57 (10.9)	23 (40.5)	22 (38.5)	3 (5.7)	9 (15.4)	24 (41.1)	20 (34.3)	3 (5.0)	11 (19.6)
	Midwest	87 (16.6)	36 (40.8)	22 (24.9)	16 (18.5)	14 (15.8)	24 (27.6)	32 (37.2)	14 (16.3)	17 (18.9)
	South	213 (40.6)	93 (43.5)	64 (30.1)	33 (15.3)	24 (11.1)	71 (33.2)	71 (33.1)	23 (10.6)	49 (23.1)
	West	168 (31.9)	77 (45.8)	45 (27.1)	15 (9.0)	30 (18.1)	50 (29.9)	59 (35.2)	19 (11.2)	40 (23.7)
Rao-Scott chi-s quared p			0.885	0.411	0.080	0.360	0.437	0.390	0.310	0.828
**Age** (years)	21–29	149 (28.3)	46 (31.0)	62 (41.4)	16 (11.0)	25 (16.6)	31 (20.9)	72 (48.4)	9 (5.9)	37 (24.8)
	30–44	280 (53.1)	130 (46.5)	68 (24.3)	41 (14.7)	40 (14.5)	97 (34.8)	82 (29.3)	40 (14.2)	61 (21.7)
	≥45	97 (18.5)	52 (53.8)	24 (24.4)	10 (9.8)	12 (11.9)	40 (41.3)	28 (28.6)	10 (10.3)	19 (19.9)
Rao-Scott chi-s quared p			**0.002**	**0.003**	0.402	0.654	**0.004**	**0.001**	0.054	0.676
**Age of first use of ENDS**	<21	107 (20.5)	35 (32.5)	45 (41.8)	9 (8.8)	18 (16.8)	20 (18.9)	52 (48.4)	6 (5.5)	29 (27.1)
	≥21	417 (79.5)	194 (46.5)	109 (26.1)	58 (13.8)	57 (13.7)	149 (35.6)	130 (31.2)	52 (12.6)	86 (20.6)
Rao-Scott chi-s quared p			**0.022**	**0.004**	0.220	0.440	**0.003**	**0.003**	**0.038**	0.194
Gender*	Man	302 (57.5)	131 (43.4)	93 (30.7)	36 (11.8)	43 (14.1)	94 (30.9)	115 (38.0)	35 (11.5)	59 (19.6)
	Woman	218 (41.4)	95 (43.4)	58 (26.6)	31 (14.4)	34 (15.6)	74 (34.1)	66 (30.3)	24 (10.9)	54 (24.7)
	Non-man/woman	4 (0.7)	2 (50.0)	2 (50.0)	0 (0)	0 (0)	1 (25.0)	0 (0)	0 (0)	3 (75.0)
	Prefer not to answer	2 (0.4)	1 (50.0)	1 (50.0)	0 (0)	0 (0)	0 (0)	1 (50.0)	0 (0)	1 (50.0)
Rao-Scott chi-squared p			0.987	0.325	0.424	0.654	0.470	0.079	0.840	0.193
Race/ethnicity*	Non-Hispanic White	449 (85.3)	193 (43.0)	132 (29.4)	61 (13.6)	63 (14.0)	142 (31.6)	155 (34.6)	51 (11.4)	101 (22.5)
	Hispanic White	21 (4.0)	11 (54.6)	5 (23.6)	2 (7.2)	3 (14.6)	10 (47.3)	5 (22.6)	2 (7.4)	5 (22.6)
	Non-Hispanic other single race	12 (2.2)	5 (38.2)	3 (28.2)	1 (9.3)	3 (24.4)	3 (23.2)	4 (37.2)	2 (18.0)	3 (21.6)
	Hispanic other single race	7 (1.3)	2 (35.8)	3 (49.9)	1 (14.4)	0 (0.0)	2 (27.2)	3 (49.9)	1 (8.5)	1 (14.4)
	Non-Hispanic multi-race	21 (4.1)	10 (46.1)	6 (25.8)	1 (4.8)	5 (23.4)	7 (31.3)	9 (41.2)	2 (9.0)	4 (18.5)
	Hispanic multi-race	8 (1.5)	2 (25.9)	3 (39.2)	1 (17.6)	1 (17.2)	2 (24.9)	3 (43.1)	1 (5.0)	2 (27.0)
	Prefer not to answer	8 (1.5)	5 (63.0)	1 (17.0)	0 (0.0)	2 (20.0)	4 (47.2)	2 (23.2)	1 (9.8)	2 (19.7)
Rao-Scott chi-s quared p			0.619	0.678	0.574	-	0.509	0.550	0.818	0.972
Annual household income* ($)	<40000	195 (37.2)	86 (44.0)	47 (24.1)	33 (16.9)	29 (15.0)	75 (38.3)	59 (30.3)	21 (10.9)	40 (20.5)
	40000–59999	104 (19.8)	49 (46.7)	27 (26.3)	7 (6.3)	22 (20.7)	22 (21.0)	37 (35.7)	10 (10.1)	35 (33.3)
	60000–99999	119 (22.6)	48 (40.4)	44 (36.8)	13 (11.2)	14 (11.6)	37 (30.7)	45 (37.8)	16 (13.2)	22 (18.2)
	≥100000	99 (18.9)	43 (43.0)	33 (33.4)	13 (13.4)	10 (10.3)	33 (33.4)	38 (38.2)	11 (10.8)	17 (17.5)
	Prefer not to answer	8 (1.5)	3 (41.6)	2 (26.0)	1 (9.5)	2 (23.0)	2 (28.9)	3 (31.4)	0 (0)	3 (39.8)
Rao-Scott chi-s quared p			0.873	0.141	0.110	0.221	0.057	0.544	0.916	**0.042**
Sexual orientation*	Non-heterosexual	112 (21.3)	40 (35.9)	39 (34.7)	9 (8.1)	24 (21.3)	28 (24.7)	47 (41.8)	5 (4.1)	33 (29.4)
	Heterosexual or straight	397 (75.5)	178 (44.8)	113 (28.5)	54 (13.6)	52 (13.1)	136 (34.2)	131 (33.0)	51 (12.9)	79 (20.0)
	Prefer not to answer	17 (3.2)	10 (62.2)	1 (8.3)	4 (24.6)	1 (4.8)	5 (32.8)	4 (24.8)	2 (14.1)	5 (28.3)
Rao-Scott chi-squared p			0.123	0.239	0.146	**0**.049	0.076	0.110	**0.004**	**0.048**

The numbers of participants reported in the table were weighted sample numbers scaled to the unweighted sample size. Due to rounding of the rescaled numbers, the sum of the column totals may not be exactly the same as the effective sample size.

* The Rao-Scott chi-squared tests were conducted after excluding the ‘prefer not to answer’ responses.

**Table 2:** Smoking status, device-liquid grouping and ENDS dependence by primary reasons for continued ENDS usage, VAPER cohort study, wave 2 (December 2020–April 2021) and wave 5 (February–April 2023) (N=526)

		*W2 n (row %)*	*W2: Reasons for continued ENDS usage*				*W5 n (row %)*	*W5: Reasons for continued ENDS usage*			
			*Quitting/reducing smoking*	*Addiction*	*Flavor*	*Others*		*Quitting/reducing smoking*	*Addiction*	*Flavor*	*Others*
			*n (row %)*	*n (row %)*	*n (row %)*	*n (row %)*		*n (row %)*	*n (row %)*	*n (row %)*	*n (row %)*
**Total**		526 (100)	229 (43.5)	154 (29.2)	67 (12.8)	77 (14.6)	526	169 (32.1)	182 (34.6)	58 (11.1)	117 (22.2)
**Device-liquid grouping**	Disposable device (salt, no adj. settings)	41 (7.9)	10 (24.1)	17 (43.0)	3 (7.6)	10 (25.3)	132 (25.2)	37 (27.7)	60 (45.1)	10 (7.3)	26 (19.8)
	Disposable pod (salt, no adj. settings)	96 (18.6)	41 (43.0)	34 (35.5)	2 (2.5)	18 (19.0)	69 (13.2)	22 (32.1)	33 (46.9)	2 (2.5)	13 (18.5)
	Refillable pod (freebase, adj. settings)	72 (13.8)	34 (48.0)	18 (24.8)	9 (12.3)	11 (14.9)	62 (11.8)	22 (35.0)	17 (27.1)	8 (12.4)	16 (25.5)
	Refillable pod (salt, adj. settings)	95 (18.4)	40 (42.4)	38 (39.7)	12 (12.6)	5 (5.4)	68 (13.0)	14 (20.6)	28 (40.9)	5 (6.9)	22 (31.6)
	Refillable tank (freebase, adj. settings)	194 (37.5)	94 (48.7)	37 (19.2)	38 (19.5)	24 (12.6)	144 (27.4)	53 (37.2)	31 (21.9)	25 (17.4)	34 (23.5)
	Others	19 (3.7)	6 (33.4)	5 (28.2)	1 (2.1)	7 (36.3)	49 (9.3)	21 (42.3)	14 (27.7)	8 (16.1)	7 (13.9)
Rao-Scott c hi-squared p			0.135	**0.004**	**0.0005**	**0.005**		0.169	**0.001**	**0.023**	0.343
**Smoking status**	Have never smoked	70 (13.3)	13 (19.1)	27 (39.2)	14 (19.8)	15 (21.9)	63 (12.0)	4 (6.2)	36 (57.7)	5 (7.4)	18 (28.6)
	Formerly smoked	339 (64.6)	153 (45.0)	104 (30.6)	41 (12.2)	41 (12.1)	366 (69.8)	107 (29.1)	129 (35.3)	49 (13.4)	81 (22.1)
	Currently smoke	116 (22.1)	63 (53.8)	22 (19.1)	12 (10.2)	20 (16.9)	96 (18.2)	57 (59.1)	16 (17.2)	5 (4.9)	18 (18.8)
Rao-Scott chi-squared p			**<0.001**	**0.021**	0.215	0.102		**<0.001**	**<0.001**	**0.030**	0.422
**ENDS dependence** (range: 0–4), mean (SE)		2.3 (0.0)	2.1 (0.1)	2.8 (0.1)	2.1 (0.1)	2.1 (0.1)	2.5 (0.0)	2.2 (0.1)	2.9 (0.1)	2.0 (0.1)	2.4 (0.1)
T-test p			**<0.0001**	**<0.0001**	**0.019**	**0.007**		**<0.0001**	**<0.0001**	**<0.0001**	0.192

The numbers of participants reported in the table were weighted sample numbers scaled to the unweighted sample size. Due to rounding of the rescaled numbers, the sum of the column totals may not be exactly the same as the effective sample size. SE: standard error.

### Factors associated with primary reason for continued ENDS usage in waves 2 and 5

The primary reason for continued ENDS usage was significantly associated with age, age of first ENDS use, smoking status, and device-liquid grouping. In wave 2, participants who were younger (<30 years vs ≥45 years) or first used ENDS aged <21 years were less likely to report quitting or cutting down smoking as their primary reason for use (31.0% vs 53.8%, p=0.002; 32.5% vs 46.5%, p=0.022, respectively); and more likely to report addiction as the reason (41.4% vs 24.4%, p=0.003; 41.8% vs 26.1%, p=0.004, respectively) compared to their counterparts (Table 1). The age difference in the primary reason for continued ENDS usage may relate to their smoking status. Nearly all participants aged ≥45 years either currently or formerly smoked, which was significantly higher compared to participants aged <30 years (98.4% vs 75.0%, p<0.0001). Participants who have never smoked were more likely to report addiction as their primary reason for continued usage compared to those who formerly smoked or currently smoke (48.0% vs 28.2% vs 21.2%, respectively, p=0.003) (Table 2). Participants reporting addiction as the primary reason had significantly higher ENDS dependence compared to participants reporting other reasons (2.8 vs 2.1, p<0.0001). These significant associations were also found in wave 5 (Tables 1 and 2).

### Associations between primary reasons for continued ENDS usage and device-liquid grouping

A set of multivariable logistic regression models was utilized to examine the associations between the four primary reasons for continued ENDS usage (quitting/reducing smoking, addiction, flavor, other reasons) and device-liquid grouping in each wave after controlling for age, race, sexual orientation, and smoking status (Table 3). Compared to those using tanks (freebase, adjustable settings), participants using disposable devices (salt, no adjustable settings) were less likely to report quitting or reducing smoking as the primary reason (W2: AOR=0.34; 95% CI: 0.14–0.80; W5: AOR=0.52; 95% CI: 0.28–1.00); and participants using device-liquid groupings with a nicotine salt liquid were more likely to report addiction as the primary reason (disposable device [salt, no adjustable settings]: W2: AOR=2.91; 95% CI: 1.33–6.36, W5: AOR=3.50; 95% CI: 1.82–6.73; disposable pod [salt, no adjustable settings]: W2: AOR=2.15; 95% CI: 1.12–4.15, W5: AOR=3.89; 95% CI: 1.85–8.19; refillable pod [salt, adjustable settings]: W2: AOR=2.47; 95% CI: 1.26–4.82, W2: AOR=2.67; 95% CI: 1.25–5.70). Regarding flavor as the primary reason for continued usage, participants using disposable devices (salt, no adjustable settings) were less likely to report flavor as the primary reason in wave 2 (AOR=0.12; 95% CI: 0.03–0.44).

**Table 3:** Factors associated with primary reasons for continued ENDS usage, VAPER cohort study, wave 2 (December 2020–April 2021) and wave 5 (February–April 2023) (N=465)

*Variables*	*Reasons for continued ENDS usage*							
	*Model 1: Quitting/reducing smoking*		*Model 2: Addiction*		*Model 3: Flavor*		*Model 4: Other reasons*	
	*W2 (N=465)*	*W5 (N=452)*	*W2 (N=465)*	*W5 (N=452)*	*W2 (N=465)*	*W5 (N=452)*	*W2 (N=465)*	*W5 (N=452)*
	*AOR (95% CI)*	*AOR (95% CI)*	*AOR (95% CI)*	*AOR (95% CI)*	*AOR (95% CI)*	*AOR (95% CI)*	*AOR (95% CI)*	*AOR (95% CI)*
**Device-liquid grouping**								
**Refillable tank** ® (freebase, adj. settings)	1		1		1		1	
Disposable device (salt, no adj. settings)	**0.34 (0.14–0.80)**	**0.52 (0.28–0.995)**	**2.91 (1.33–6.36)**	**3.50 (1.82–6.73)**	0.36 (0.11–1.21)	0.41 (0.16–1.09)	1.96 (0.68–5.64)	0.77 (0.39–1.52)
Disposable pod (salt, no adj. settings)	0.85 (0.47–1.54)	0.64 (0.31–1.32)	**2.15 (1.12–4.15)**	**3.89 (1.85–8.19)**	**0.12 (0.03–0.44)**	0.14 (0.02–1.14)	1.41 (0.67–3.00)	0.72 (0.32–1.60)
Refillable pod (freebase, adj. settings)	0.82 (0.42–1.59)	0.61 (0.27–1.36)	1.46 (0.69–3.11)	1.99 (0.90–4.39)	0.68 (0.27–1.68)	0.86 (0.33–2.25)	1.27 (0.54–2.98)	0.93 (0.38–2.25)
Refillable pod (salt, adj. settings)	0.91 (0.49–1.67)	0.43 (0.17–1.08)	**2.47 (1.26–4.82)**	**2.67 (1.25–5.70)**	0.58 (0.24–1.38)	0.32 (0.10–1.05)	0.34 (0.11–1.07)	1.34 (0.61–2.97)
**Age**	**1.47 (1.05–2.06)**	**1.45 (1.00–2.09)**	0.71 (0.49–1.03)	0.75 (0.53–1.05)	0.92 (0.58–1.49)	1.10 (0.61–2.00)	0.89 (0.57–1.39)	0.93 (0.62–1.40)
**Race**								
White ®	1		1		1		1	
Other single race	1.07 (0.44–2.57)	0.90 (0.30–2.72)	1.16 (0.52–2.56)	0.82 (0.35–1.91)	0.63 (0.16–2.52)	1.92 (0.61–6.06)	1.03 (0.39–2.72)	0.83 (0.33–2.11)
Multi-race	0.99 (0.51–1.95)	0.81 (0.37–1.73)	1.00 (0.51–1.96)	1.34 (0.65–2.77)	0.56 (0.17–1.84)	1.09 (0.38–3.14)	1.41 (0.62–3.20)	0.86 (0.42–1.76)
**Sexual orientation**								
Non-heterosexual ®	1		1		1		1	
Heterosexual or straight	1.37 (0.81–2.30)	1.46 (0.79–2.70)	0.83 (0.49–1.39)	0.85 (0.50–1.44)	1.73 (0.73–4.13)	**3.07 (1.10–8.55)**	0.58 (0.30–1.12)	0.61 (0.35–1.08)
**Smoking status**								
Currently smoke ®	1		1		1		1	
Have never smoked	**0.19 (0.09–0.42)**	**0.05 (0.01–0.16)**	**2.69 (1.21–6.00)**	**6.77 (2.88–15.93)**	2.27 (0.76–6.74)	1.99 (0.46–8.63)	1.33 (0.53–3.34)	1.56 (0.62–3.90)
Formerly smoked	0.65 (0.38–1.11)	0.22 (0.12–0.40)	**2.10 (1.10–4.03)**	**3.36 (1.71–6.60)**	1.09 (0.46–2.62)	**3.33 (1.16–9.51)**	0.71 (0.34–1.48)	1.19 (0.58–2.45)
AIC	736088.44	594968.20	659666.82	646295.21	403621.55	328613.59	440200.86	580968.96
SC	736212.35	595091.56	659790.73	646418.57	403745.47	328736.95	440324.78	581092.32
–2 Log L	736066.44	594946.20	659644.82	646273.21	403599.55	328591.59	440178.86	580946.96

The table reported results of multivariable logistic regression models. The numbers of participants reported in the table were weighted sample numbers scaled to the unweighted sample size. Due to rounding of the rescaled numbers, the sum of the column totals may not be exactly the same as the effective sample size. AOR: adjusted odds ratio. ® Reference categories.

**Figure 1:**
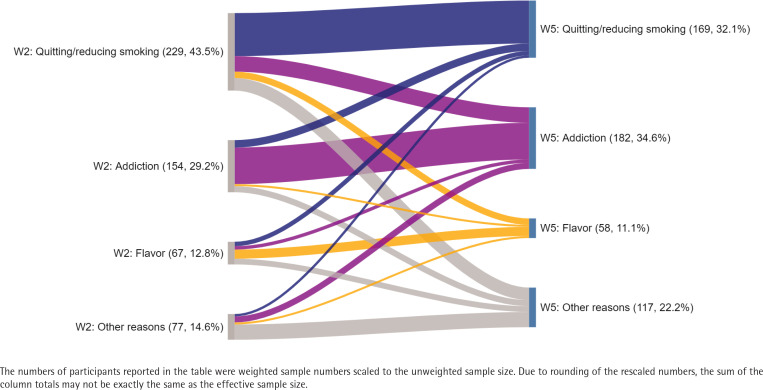
Change in primary continued ENDS usage reasons from wave 2 to 5, VAPER cohort study, wave 2 (December 2020–April 2021) and wave 5 (February–April 2023) (N=526)

From wave 2 to 5, there was a significant change in the primary reason for continued ENDS usage, with fewer participants reporting quitting or cutting down smoking (43.5% vs 32.1%, p<0.001) and more reporting addiction (29.2% vs 34.6%, p=0.014) (Figure 1). The percentage of people reporting flavor as the primary reason remained nearly the same (12.8% vs 11.1%, p=0.292). An increase in participants using disposable devices (salt, no adjustable settings) (7.9% vs 25.2%) was also observed. At the same time, there was a decrease in using tanks (freebase, adjustable settings) (37.5% vs 27.4%), disposable pods (salt, no adjustable settings) (18.6% vs 13.2%), refillable pods (salt, adjustable settings) (18.4% vs 13.0%), and refillable pods (freebase, adjustable settings) (13.8% vs 11.8%) (Table 2). The association between the change in the primary reason for continued ENDS usage and device-liquid grouping was explored. Among participants who reported quitting/reducing smoking or addiction as the primary reason in wave 2, more than half reported the same reason in wave 5 (55.7% and 70.4%, respectively). Compared to those who changed from addiction to other reasons, participants who consistently reported addiction as their primary reason for continued ENDS usage were less likely to use tanks (freebase, adjustable settings) (14.2% vs 41.9%) and more likely to use disposable pods (salt, no adjustable settings) in wave 5 (18.9% vs 6.2%) (p=0.010) (Table 4). Compared to those changing from flavor to other reasons, a greater proportion of participants who consistently reported flavor as the reason, used tanks (freebase, adjustable settings) in wave 5 (50.6% vs 31.1%) and a smaller proportion used disposable devices (salt, no adjustable settings) (6.2% vs 19.8%) or refillable pods (salt, adjustable settings) (5.9% vs 26.2%). However, the difference was not statistically significant (p=0.087).

**Table 4:** Change in primary reasons for continued ENDS usage by device-liquid grouping in wave 5, VAPER cohort study, wave 2 (December 2020–April 2021) and wave 5 (February–April 2023) (N=526)

*Device-liquid grouping in W5*	*Quitting/reducing smoking in W2*		*Addiction in W2*		*Flavor in W2*		*Others in W2*	
	*No change in W5*	*Change in W5*	*No change in W5*	*Change in W5*	*No change in W5*	*Change in W5*	*No change in W5*	*Change in W5*
	*n (%)*	*n (%)*	*n (%)*	*n (%)*	*n (%)*	*n (%)*	*n (%)*	*n (%)*
Total	127 (55.7)	101 (44.3)	108 (70.4)	45 (29.6)	27 (40.9)	40 (59.1)	45 (59.1)	31 (40.9)
Disposable device (salt, no adj. settings)	25 (19.8)	25 (24.2)	39 (36.0)	13 (28.9)	2 (6.2)	8 (19.8)	12 (25.7)	9 (28.8)
Disposable pod (salt, no adj. settings)	17 (13.1)	9 (9.3)	20 (18.9)	3 (6.2)	2 (6.7)	4 (10.3)	9 (19.5)	5 (17.0)
Refillable pod (freebase, adj. settings)	19 (15.0)	15 (14.7)	9 (8.2)	2 (3.5)	4 (17.2)	3 (8.7)	4 (9.5)	5 (16.8)
Refillable pod (salt, adj. settings)	11 (8.3)	11 (10.4)	18 (16.3)	4 (9.8)	2 (5.9)	10 (26.2)	7 (15.6)	6 (19.1)
Refillable tank (freebase, adj. settings)	39 (30.7)	32 (31.3)	15 (14.2)	19 (41.9)	13 (50.6)	12 (31.1)	12 (26.1)	2 (4.9)
Others	17 (13.1)	10 (10.1)	7 (6.3)	4 (9.7)	3 (13.6)	2 (3.9)	2 (3.5)	4 (13.4)
Rao-Scott chi-squared p	0.909		**0.010**		0.087		0.178	

The numbers of participants reported in the table were weighted sample numbers scaled to the unweighted sample size. Due to rounding of the rescaled numbers, the sum of the column totals may not be exactly the same as the effective sample size.

## DISCUSSION

This study examined the association between the primary reason for continued ENDS usage and device and liquid characteristics. We explored changes in the primary reason among US adults frequently using ENDS from December 2020 to April 2023. The study suggested that the primary reasons for continued ENDS usage varied significantly by age, age of first use of ENDS, smoking status, and the combinations of ENDS device type, device adjustability, and liquid formulation in each wave. We also found a significant increase in the proportion of people continuing to use ENDS primarily due to addiction. In contrast, a significant decrease was observed in those using ENDS to quit or reduce smoking.

Quitting or cutting down smoking was the most common reason for continued ENDS usage in wave 2 (December 2020–April 2021), with 43.5% of people reporting this as the primary reason. However, it significantly declined to 32.1% in wave 5 (January–April 2023). Although a lower proportion of participants smoked in wave 5, the data suggest that people’s preferences for ENDS types may be associated with motivations toward quitting or reducing smoking. In this study, individuals using tanks (freebase, adjustable settings) had higher odds of using ENDS for quitting or reducing smoking compared to those using disposable devices (salt, no adjustable settings) in both waves. Additionally, we observed that people who were older or currently smoked were more likely to be motivated to use ENDS to quit or reduce smoking. Regulations on tanks (freebase, adjustable settings) may have an outsized impact on people using ENDS to quit or reduce smoking, while regulations on disposable devices (salt, no adjustable settings) may have a lesser impact on this group. Our findings are consistent with some previous studies. Glasser et al.^19^ analyzed the 2013–2016 Population Assessment of Tobacco and Health data and found that using a refillable (vs disposable) ENDS was associated with an increased likelihood of quitting. Lee et al.^13^ using 2018–2019 TUS-CPS data, found that about one-third of US adult dual users used ENDS to help quit smoking. Those using disposable ENDS were less likely to use ENDS to help quit smoking compared to adults using tanks^13^. The device’s flexibility in use may contribute to users’ success in quitting smoking since it allows people to adjust nicotine intake from their devices^20^. And it is plausible that the higher cost of tanks compared to disposable devices may indicate that individuals investing in these devices are more committed to quitting smoking and more likely to use them consistently^21^. However, it is important to note that a study suggests that people using disposable devices had higher odds of reporting a past-year quit attempt than people using tanks^22^. Further research is necessary to ascertain the reasons behind these findings.

In this study, there was a significant increase in reporting addiction as the primary reason for continued frequent ENDS usage. Although fewer participants smoking in wave 5 may contribute to the switch from quitting/reducing smoking to addiction, the switch may also partially relate to the significant increase in the use of disposable devices (from 41 in W2 to 132 in W5). Disposable devices employ a high-strength nicotine salt liquid (e.g. Puff Bar with nicotine concentrations ranging from 40.6 mg/mL to 52.4 mg/mL)^23^. And participants who consistently reported addiction as their primary reason for continued ENDS usage had higher odds of using disposable pods (salt, no adjustable settings) compared to those who changed from addiction to any other reasons. A liquid with a higher concentration of nicotine may enhance nicotine absorption and may be linked to increased dependence on ENDS^24^. Earlier research suggested that young people using high-nicotine salt-based ENDS were more likely to report perceived addiction to vaping^25,26^. We also found that people using devices with a nicotine salt liquid had higher odds of reporting addiction as the primary reason for continued ENDS usage compared to people using adjustable tanks with a freebase liquid. Furthermore, people reporting addiction as the primary reason for ENDS usage had significantly higher ENDS dependence compared to people reporting other reasons. Given the perceived addictiveness of high-nicotine salt-based ENDS^27^, restrictions on disposable devices and nicotine salts may disproportionately impact those who use ENDS because of addiction.

About 10% of people reported flavor as the primary reason for ENDS usage in this study, which was relatively stable across waves. Less than 3% of people using disposable pods (salt, no adjustable settings) continued to use ENDS because of flavor in both waves, which is the lowest compared to people using other device-liquid groupings. This is likely connected to the FDA’s policy, which precedes enforcing regulations against all flavors in disposable pod-based ENDS devices, excluding menthol and tobacco. This policy does not cover flavored disposable devices and liquids utilized in tanks or refillable pods^28^. Among those attracted by flavors, a flavor ban may discourage using ENDS.

This study presents longitudinal data from a recent sample across the US that reflects ENDS devices and liquids people use and their motivations for continued ENDS usage over the years under the rapidly evolving ENDS market and recent regulations. Additionally, to enhance representativeness, post-stratification weights were implemented. Various data integrity procedures were implemented, and both photographic and self-report data for liquids were utilized to optimize the data quality^16^.

### Limitations

This study should be interpreted within the context of its limitations. First, participants in this study used ENDS ≥5 days per week in both waves. Thus, findings cannot be generalized to a population with less frequent ENDS usage or a population using ENNDS. The sample could be biased if individuals who successfully quit smoking using ENDS then reduced or stopped using them (<5 days/week). Second, to maintain a manageable survey length for participants, detailed device and liquid data were collected and reported only for participants’ most commonly used device and liquid, not accounting for other devices and liquids they might use. Third, we grouped ‘To use when I’m not allowed to smoke’, ‘Absence of smell’, ‘Because I enjoy the boost’, ‘Curiosity/Just want to try them’, and ‘Some other reason (including stress, anxiety, depression, or other mental health concerns, etc.)’ together as ‘Other reasons’ for continued ENDS usage due to the small sample size of these options and analysis purposes. Results of ‘Other reasons’ provide limited information to inform regulatory efforts. And participants self-reported the primary reason for continued ENDS usage. There could be some reporting bias. In addition, the retention rate for wave 2 to wave 5 is 44.3%. Although there were no significant differences in sociodemographic characteristics between the study sample and other participants in wave 2 who did not respond to wave 5 survey, there could be a potential attrition bias.

## CONCLUSIONS

Over 2.5 years, more people continued to use ENDS at least five days per week primarily because of addiction and less because of quitting or reducing smoking. Reasons for continued ENDS usage vary significantly depending on the combination of ENDS device type, device adjustability, and liquid formulation. The findings of this study can enhance understanding of the primary motivations of continued ENDS usage and suggest that regulatory efforts on tanks with freebase liquid may disproportionately impact individuals using ENDS for smoking cessation or reduction. In contrast, restrictions on disposable devices and nicotine salts may disproportionately impact those using ENDS due to addiction.

## Data Availability

The data supporting this research are available from the authors on reasonable request.
